# Shared genetic etiology between ADHD, task-related behavioral measures and brain activation during response inhibition in a youth ADHD case–control study

**DOI:** 10.1007/s00406-023-01632-8

**Published:** 2023-06-28

**Authors:** Gülhan Saraçaydın, I. Hyun Ruisch, Daan van Rooij, Emma Sprooten, Barbara Franke, Jan K. Buitelaar, Andrea Dietrich, Pieter J. Hoekstra

**Affiliations:** 1 Department of Child and Adolescent Psychiatry, University Medical Center Groningen, University of Groningen, Hanzeplein 1, 9713 GZ Groningen, The Netherlands; 2 Accare Child Study Center, Groningen, The Netherlands; 3 Donders Institute for Brain, Cognition and Behavior, Radboud University, Nijmegen, The Netherlands; 4 Departments of Psychiatry and Human Genetics, Radboud University Medical Center, Nijmegen, The Netherlands

**Keywords:** Attention-deficit/hyperactivity disorder, Polygenic risk score, fMRI, Response inhibition, Stop-signal task

## Abstract

Impaired response inhibition is commonly present in individuals with attention-deficit/hyperactivity disorder (ADHD) and their unaffected relatives, suggesting impaired response inhibition as a candidate endophenotype in ADHD. Therefore, we explored whether behavioral and neural correlates of response inhibition are related to polygenic risk scores for ADHD (PRS-ADHD). We obtained functional magnetic resonance imaging of neural activity and behavioral measures during a stop-signal task in the NeuroIMAGE cohort, where inattention and hyperactivity-impulsivity symptoms were assessed with the Conners Parent Rating Scales. Our sample consisted of 178 ADHD cases, 103 unaffected siblings, and 173 controls (total *N* = 454; 8–29 years), for whom genome-wide genotyping was available. PRS-ADHD was constructed using the PRSice-2 software. We found PRS-ADHD to be associated with ADHD symptom severity, a slower and more variable response to Go-stimuli, and altered brain activation during response inhibition in several regions of the bilateral fronto-striatal network. Mean reaction time and intra-individual reaction time variability mediated the association of PRS-ADHD with ADHD symptoms (total, inattention, hyperactivity-impulsivity), and activity in the left temporal pole and anterior parahippocampal gyrus during failed inhibition mediated the relationship of PRS-ADHD with hyperactivity-impulsivity. Our findings indicate that PRS-ADHD are related to ADHD severity on a spectrum of clinical, sub-threshold, and normal levels; more importantly, we show a shared genetic etiology of ADHD and behavioral and neural correlates of response inhibition. Given the modest sample size of our study, future studies with higher power are warranted to explore mediation effects, suggesting that genetic liability to ADHD may adversely affect attention regulation on the behavioral level and point to a possible response inhibition-related mechanistic pathway from PRS-ADHD to hyperactivity-impulsivity.

**Electronic supplementary material:**

The online version of this article (10.1007/s00406-023-01632-8) contains supplementary material, which is available to authorized users.

## Introduction

Attention-deficit/hyperactivity disorder (ADHD) is a common neurodevelopmental disorder, affecting 5–7% of children/adolescents worldwide [1, 2]. It is characterized by an age-inappropriate, persistent pattern of inattention, and/or hyperactivity and impulsive behaviors that interferes with functioning and development [3]. ADHD is a multifactorial disorder, with both genetic and environmental factors, as well as their interaction, contributing to its etiology [4–6].

One of the most prominent neurocognitive biomarkers of ADHD is impaired response inhibition, which refers to the ability to voluntarily stop or suppress behaviors that are inappropriate for the context and/or individual goals [7, 8]. Functional magnetic resonance imaging (fMRI) studies on response inhibition have shown that individuals with ADHD exhibit decreased activation during action cancellation and restraint compared with controls in specific brain areas: the fronto-parietal network, consisting of the prefrontal and superior parietal regions, and the fronto-striatal network, involving the prefrontal cortex and basal ganglia [9–15]. By focusing on action cancellation assessed by the stop-signal task [16], previous research has also reported that impairments in behavioral performance [14–17] and aberrant neural activity associated with response inhibition [14] are not only present in children and adolescents with ADHD but also in their unaffected first-degree relatives. This led researchers to propose impaired inhibitory control as a possible endophenotype or candidate neurocognitive biomarker of ADHD that shares familial loading with the phenotype [18].

Family-based studies have indeed shown that both behavioral performance of response inhibition and response inhibition-related brain activity are heritable, up to 60%. Two twin studies reported significant contribution of additive genetic variance to action cancellation during the stop-signal task [19, 20]. There has only been one genome-wide association study (GWAS) regarding behavioral response inhibition performance as assessed by the stop-signal task, but no significant loci were detected, probably due to the small sample size of 4,611 participants from a general population cohort [21]. As for the neural correlates, there is no functional magnetic resonance imaging (fMRI) study directly investigating the heritability of response inhibition-related brain activation, but two twin studies addressing action restraint using a Go/NoGo task also reported that 50 to 60% of the variance in amplitudes of response inhibition-related-event-related potential components in adolescents and adults are attributable to genetic factors [22, 23].

ADHD is a heritable disorder with a highly polygenic nature involving the combined effect of many genetic variants with small individual effects on the overall disease risk. The largest published GWAS of ADHD to date (comprising 20,183 cases and 35,191 controls) reported a SNP heritability of 22% and identified twelve genome-wide significant loci across the genome [24]. This GWAS of ADHD not only confirms the polygenic architecture of ADHD [24], but also enables the construction of polygenic risk scores (PRS) for ADHD to investigate a potentially shared genetic etiology between ADHD and cognitive and neural measures [25]. A systematic review of the existing literature on PRS-ADHD revealed that PRS-ADHD has also been linked with ADHD traits, other externalizing behaviors, impaired working memory, and reduced brain volume [26]. The relation between PRS-ADHD and hyperactivity-impulsivity symptoms may be partially mediated by neuroanatomical variation [27]. Moreover, inhibitory control (as assessed by the Stroop task) has been linked with PRS-ADHD and was found to partially mediate the link between PRS-ADHD and symptoms of ADHD [28]. A recent study investigating whether PRS-ADHD influenced attention regulation and response inhibition in ADHD reported significant associations of PRS-ADHD with reaction time variability but not with the number of commission errors during the Go/No-Go task [29]. The aforementioned studies point to genetic sharing between ADHD and different cognitive traits and neuroimaging-derived variables and also suggest that certain (response inhibition-related) cognitive and neural processes mediate the link between genetic liability to ADHD as reflected in PRS-ADHD and ADHD symptomatology. However, to date, a possible shared genetic background between ADHD and brain activation during response inhibition has not been investigated yet.

Therefore, in the present study, we investigated whether genetic liability to ADHD (PRS-ADHD) was associated with neural activity related to response inhibition (i.e., as measured during a stop-signal task), and whether such response inhibition-related neural activity would mediate the link between PRS-ADHD and ADHD symptoms. Moreover, we aimed to expand previous evidence pointing to genetic sharing between ADHD and inhibitory control [29] by investigating whether PRS-ADHD would be related to behavioral performance measures during a stop-signal task, as well as investigating a possible mediating role of these behavioral correlates in the relation between PRS-ADHD and ADHD symptoms, in a relatively modest sample of individuals with ADHD, their unaffected siblings, and controls (NeuroIMAGE).

## Methods

### Participants

All subjects participated in the NeuroIMAGE project, which is a Dutch follow-up of the International Multicenter ADHD Genetics (IMAGE) project, a multi-site international cohort study [30]. Participants were included as ADHD probands if they met criteria for ADHD diagnosis on the Kiddie Schedule for Affective Disorders and Schizophrenia Present and Lifetime Version [31] and/or Conners’ ADHD questionnaires, as obtained briefly prior to scanning [32, 33]. Inclusion criteria for unaffected siblings and controls (without an ADHD diagnosis) were having fewer than three symptoms on both inattention and hyperactivity-impulsivity subscales [30]. Details regarding the NeuroIMAGE project and exclusion criteria are provided in Supplementary Information. Participants with ADHD who used ADHD medication discontinued their medication for 24 h prior to scanning. Written informed consent was obtained from parents and from participants who were older than 12 years, in accordance with national legislation. The study had been approved by the respective local ethics committees.

Genetic data and data regarding symptoms of ADHD were available from 952 participants, but 44 participants were excluded from further analysis because of being outliers in their genetic background (see below, Genotyping). Of the remaining 908 participants (43% female, mean age = 16.9 years, see Supplementary Table 1), stop-signal task fMRI data were available from 454 participants.

### ADHD symptoms

For each participant, the severity of ADHD symptoms on the ‘cognitive problems/inattention’ (12 items) and ‘hyperactivity’ (9 items) subdomains were assessed using a parent questionnaire (Conners’ Parent Rating Scales-Revised:Long version, CPRS-R:L) [32] rated on a 4-point Likert scale from 0 (not at all) to 3 (severely). The CPRS-R:L has been shown to have high internal consistency (Cronbach’s α ranging from 0.75 to 0.94) and construct validity to discriminate individuals with ADHD from a non-clinical group; sensitivity 92.3%, specificity 94.5%, positive predictive power 94.4%, negative predictive power 92.5%) [32]. The total ADHD symptom severity score was calculated as the sum of severity scores for inattention and hyperactivity-impulsivity.

### Genotyping

Genotyping was done using the Infinium PsychArray-24 BeadChip v1.1, Illumina, comprising ~ 593 K markers. Quality control and imputation were performed using the Ricopili (Rapid Imputation for COnsortias PIpeLIne for GWAS) pipeline [34] and 1 KG phase 3 European reference samples [35]. Details regarding preprocessing and quality control of genotype data can be found in Supplementary Information. Only SNPs passing quality control filters regarding imputation quality (> 0.8), minor allele frequency (≥ 0.05), Hardy–Weinberg equilibrium test (*p*-value cut-off 1 × 10^–6^), and SNP-call rate (> 0.98) were retained. After imputation, genome-wide genotype data were available for 2,840,886 SNPs.

Four principal components were used as covariates to correct for ancestry. A scatterplot of the first and second principal components showed that the individuals from the NeuroIMAGE clustered closely with the European British and CEPH populations of the 1000 Genomes Project [35] (Supplementary Fig. 1). Individuals with values >  ± 2 SD from the mean on the first four principal components were removed (N = 44), leaving 908 subjects for the polygenic risk score analysis.

### Polygenic risk scoring

The 2019 GWAS meta-analysis for ADHD conducted by the Psychiatric Genomics Consortium (PGC) [24] was used as ‘base dataset’ to calculate individual-level PRS in NeuroIMAGE as the ‘target sample’. To avoid overlap between base and target samples, we used GWAS results in which the individuals from NeuroIMAGE were excluded. A total of 2,175,131SNPs overlapped between the base and target datasets and were available for computing PRS. PRS-ADHD were calculated using the PRSice-2 software (https://www.prsice.info) [36]. The SNPs were clumped based on linkage disequilibrium with a cutoff of *r*^2^ = 0.1 in a 250-kb bidirectional window to keep a set of independent SNPs (resulting in a total of 66,978 clumped SNPs). PRS-ADHD were initially computed for a few increasingly inclusive SNP *p*-value thresholds (*p* < 1 × 10^–6^, 1 × 10^–4^, 0.01, 0.05, 0.1, 0.2, 0.3, 0.4, 0.5, 1). From these, only the PRS-ADHD showing the strongest association with ADHD symptom scores were used in relation to the subsequent neuroimaging analyses.

### Stop-signal task

A visual version of the stop-signal task was used to probe the behavioral and neural mechanisms of response inhibition [17]. Details regarding the task are provided in the Supplementary Information. Response inhibition performance was measured by the stop-signal reaction time (SSRT), which was calculated by subtracting the mean stop-signal delay from the mean reaction time. Other task outcomes of interest were the mean reaction time to go-stimuli (MRT), and the intra-individual coefficient of variation of reaction time to go stimuli (IRT). Since MRT and IRT are related to attentional processing, these are also important (complementary) components of response inhibition, in particular when considering ADHD patients who are suffering from inattention symptomatology.

### fMRI acquisition and preprocessing

Information on imaging parameters and fMRI acquisition, and preprocessing following ICA-AROMA [37] were previously described in detail [14, 38] and can be found in the Supplementary Information.

### fMRI data analyses

#### First-level fMRI data analysis

The initial within-subject analysis was conducted across all participants using a general linear model in FSL-FEAT (FMRIB’s Software Library, www.fmrib.ox.ac.uk/fsl; fMRI Expert Analysis Tool, version 6.0) [39, 40]. Factors of interest were successful go- and successful and failed stop-trials. Failed go-trials, movement trials (trials within an 8-s interval before movements exceeding 1 mm), signal from cerebral spinal fluid and white matter, and 24 realignment parameters (six motion parameters plus their six temporal derivatives, and quadratic terms of these twelve regressors) were added as covariates. Activation maps of contrasts of interests [successful inhibition—go (using successful stop- versus successful go-trials to isolate the activation of successful inhibition and to identify brain regions that are specifically involved in response inhibition processes), failed inhibition—go (using unsuccessful stop- versus successful go-trials to identify regions that are activated when the participant fails to inhibit a prepotent response), and failed—successful inhibition (using unsuccessful versus successful stop-trials to model activation unique to the error processing and adjustment of behavior after an error)] were calculated and spatially normalized to 2-mm MNI152 template, and subsequently combined over all the runs within each subject using a fixed effects model.

#### Between-subjects fMRI data analysis

In the between-subjects analysis, mixed-effects analyses using the FSL-FLAME1 [41] procedure were conducted to generate t-contrasts with the contrasts mentioned above. As one of the main aims of the study was to identify the regions exhibiting PRS-ADHD-related activation during the stop-signal task and, according to the best of our knowledge, there has been no previous research on this, the regions of interest (ROIs) were not chosen a priori, but rather determined by hypothesis-free voxel-based analysis. We used PRS-ADHD as the regressor of interest while controlling for the above-mentioned covariates to identify the ROIs showing PRS-ADHD-related activation in three different contrasts, namely successful inhibition—go, failed inhibition—go, and failed—successful inhibition. We used the FSL default cluster-forming threshold of *Z* > 2.3 for Z-statistic images [41]—which is commonly used and should provide a sufficiently stringent cut-off to distinguish random noise from signal [14, 42–44], while a family-wise error rate (FWER)-corrected cluster significance threshold of p = 0.05 across the whole brain was applied. After the voxel-based analyses, the mean parameter estimates for all clusters found to be associated with PRS-ADHD (i.e., the identified ROIs) were extracted for each participant for further analyses outside FSL. It was not feasible to correct for sibling relatedness in FSL, but for all subsequent analyses using the mean neural activity from clusters that mapped significantly onto PRS-ADHD, we applied this correction in the regression and mediation models (see Statistical data analysis).

### Statistical data analysis

We performed a series of regression analysis based on the Baron and Kenny [45] analysis strategy to test our mediation hypotheses. A detailed explanation of the analysis strategy can be found in the Supplementary Information. All statistical analyses were performed using *R* 4.0.2 (https://www.r-project.org/) [46]. A false discovery rate (FDR) [47] correction was applied to correct for multiple testing (see Supplementary Information).

#### Regression models

Mixed model regression analyses (using R package lme4 [48]) were performed to test for separate associations between PRS-ADHD, ADHD symptom severity, and behavioral and neural correlates of response inhibition. To explore the direct effect of PRS-ADHD (predictor) on ADHD symptoms (outcome) (c’ path on Baron and Kenny’s mediation model [45]), we first investigated associations of PRS-ADHD with ADHD symptom severity (inattention, hyperactivity-impulsivity, and total ADHD severity). The *p-value* threshold at which the PRS-ADHD showed the strongest association with ADHD symptom severity was selected for the subsequent analyses. Second, to evaluate the effect of PRS-ADHD (predictor) on response inhibition-related behavioral measures and brain activation (mediator) (a path on Baron and Kenny’s mediation model [45]), we investigated association of PRS-ADHD with the Stop-Signal performance parameters (MRT, IRT, and SSRT) and the mean SSRT-associated brain activation (extracted from the clusters during successful inhibition—go, failed inhibition—go, and failed—successful inhibition fMRI contrasts); those significant after FDR-correction were selected for the next analyses. Third, to investigate the effect of response inhibition-related behavioral measures and brain activation (mediator) on ADHD symptoms (outcome) (b path on Baron and Kenny’s mediation model [45]), we explored the associations of behavioral and neural correlates of response inhibition with ADHD symptom severity, controlling for PRS-ADHD; those significant after FDR-correction were selected for mediation analyses (see section below). Mediation (indirect effect) can be estimated by the product of the a × b path coefficients. Before establishing the mediation model, this regression analysis was run to find out which mediators were associated with ADHD symptoms. Age and sex were included as fixed covariates in all analyses, and, where applicable, family identity was included as a random variable to adjust for sibling relatedness. In addition, genotyping batch and the first four genetic principal components were entered as covariates in the analyses involving PRS-ADHD, while fMRI scanning site was entered as a covariate in the analyses involving neural activity.

#### Mediation analyses

Mediation analyses (using R package mediation [49]) were performed to explore the potential mediation effect of behavioral outcomes and neural activity of the stop-signal task associated with PRS-ADHD on the relation between PRS-ADHD and ADHD symptoms. Behavioral and neural correlates of response inhibition that were found to be associated with ADHD symptoms after controlling for PRS-ADHD were selected as potential mediators. The mediation (indirect), direct, and total effects were estimated using mixed models involving family identity as a random factor and aforementioned covariates. The quasi-Bayesian Monte Carlo simulation was used with 10,000 iterations to generate 95% confidence intervals for estimates. To control Type I errors, we applied FDR correction for the behavioral and neural correlates of response inhibition separately because these are different experiments (see ‘Mediation analyses’ in the Results section).

## Results

### Participants

The main sample consisted of 454 participants with both genetic and imaging data available, originating from 267 families. Overall, 178 (39.2%) met criteria for ADHD, 103 (22.7%) were unaffected siblings, and 173 (38.1%) were controls. Details regarding sample characteristics can be found in Table 1.

**Table 1:** Demographic characteristics and stop-signal task outcome measures

Demographic characteristics				
	*N*			
Sex (female/male)	454	197 (43.4%)/ 257 (56.6%)		
Medication use (yes/no)	453	77 (17%)/ 376 (83%)		
Handedness (right/left)	451	49 (10.9%)/ 402 (89.1%)		
	N	Mean	SD	Range
Age	454	17.1	3.5	[7.7–29.2]
Estimated IQ^a^	451	100.1	16.56	[55–144]
Total ADHD symptom score^b^	423	11.98	11.78	[0–52]
Inattention symptom score^b^	438	7.73	7.21	[0–27]
Hyperactivity-impulsivity symptom score^b^	431	4.57	5.43	[0–27]
*Stop-signal task outcomes*				
MRT (ms)	454	497.9	91.61	
IRT(ms)	454	0.19	0.05	
SSRT (ms)	454	259.2	78.58	

*N*, number of participants with data available; attention-deficit/hyperactivity disorder (ADHD); *MRT* mean reaction time to go-stimuli, *IRT* intra-individual coefficient of variation of reaction time to go stimuli, *SSRT* stop-signal reaction time

^a^Based on the block-design and vocabulary subtests of the Wechsler Intelligence Scale for Children or Wechsler Adult Intelligence Scale[64]

^b^Scores on the Conners’ Parent Rating Scale—Revised: Long version[32]

### PRS-ADHD and ADHD symptoms

There were positive associations between PRS-ADHD and total ADHD, inattention, and hyperactivity-impulsivity symptom scores at all PRS *p*-value thresholds except for 1 × 10^–4^ and 1 × 10^–6^ in all participants with genetic data available (*N* = 908; Table 2). The results in the participants with both genetic and fMRI data available (*N* = 454) were similar (Supplementary Table 2). The strongest association was observed at a *p*-value threshold of 1 for total ADHD (*R*^2^-PRS = 0.044; p-FDR = 9.21 × 10^–9^), inattention (*R*^2^-PRS = 0.039; p-FDR = 1.32 × 10^–8^), and hyperactivity-impulsivity symptom scores (*R*^2^-PRS = 0.04; p-FDR = 1.65 × 10^–8^). Therefore, the PRS-ADHD at a* p*-value threshold of 1 (66,978 SNPs), which also explained the most variance for all symptom scales, was selected for further analyses.

**Table 2:** Associations between polygenic risk score for ADHD (PRS-ADHD) at a range of p-value thresholds and total, inattention, and hyperactivity-impulsivity symptom scores for all participants with genetic data available

Independent variable		Dependent variable											
		Total symptom score (*N* = 845)				Inattention symptoms (*N* = 866)				Hyperactivity-impulsivity symptoms (*N* = 859)			
PRS PT	# of SNPs	β (SE)	p-uncor	p-FDR	R^2^-PRS	β (SE)	p-uncor	p-FDR	R^2^-PRS	β (SE)	p-uncor	p-FDR	R^2^-PRS
1	66,978	0.215 (0.034)	3.07 × 10^–10^	9.21 × 10^–9^	0.044	0.202 (0.033)	1.58 × 10^–9^	1.32 × 10^–8^	0.039	0.204 (0.034)	3.14 × 10^–9^	1.65 × 10^–8^	0.04
0.5	49,274	0.209 (0.034)	1.04 × 10^–9^	1.32 × 10^–8^	0.042	0.194 (0.033)	7.53 × 10^–9^	2.51 × 10^–8^	0.036	0.199 (0.034)	8.37 × 10^–9^	2.51 × 10^–8^	0.038
0.4	43,169	0.206 (0.034)	1.76 × 10^–9^	1.32 × 10^–8^	0.041	0.188 (0.033)	2.31 × 10^–8^	5.33 × 10^–8^	0.033	0.199 (0.034)	8.29 × 10^–9^	2.51 × 10^–8^	0.038
0.3	35,937	0.202 (0.034)	3.85 × 10^–9^	1.65 × 10^–8^	0.039	0.184 (0.033)	4.66 × 10^–8^	9.99 × 10^–8^	0.032	0.195 (0.034)	1.84 × 10^–8^	4.60 × 10^–8^	0.037
0.2	27,386	0.204 (0.034)	3.35 × 10^–9^	1.65 × 10^–8^	0.04	0.18 (0.034)	1.03 × 10^–7^	1.82 × 10^–7^	0.031	0.197 (0.034)	1.31 × 10^–8^	3.57 × 10^–8^	0.038
0.1	16,756	0.185 (0.034)	8.50 × 10^–8^	1.59 × 10^–7^	0.033	0.165 (0.034)	1.12 × 10^–6^	1.68 × 10^–6^	0.026	0.176 (0.034)	3.92 × 10^–7^	6.19 × 10^–7^	0.03
0.05	10,133	0.185 (0.034)	8.48 × 10^–8^	1.59 × 10^–7^	0.033	0.163 (0.034)	1.56 × 10^–6^	2.23 × 10^–6^	0.025	0.183 (0.034)	1.55 × 10^–7^	2.58 × 10^–7^	0.033
0.01	2,898	0.125 (0.035)	3.4 × 10^–4^	4.45 × 10^–4^	0.015	0.107 (0.034)	0.002	0.003	0.011	0.126 (0.035)	3.1 × 10^–4^	4.16 × 10^–4^	0.016
1 × 10^–4^	79	-0.006 (0.035)	0.871	0.871	1 × 10^–4^	0.013 (0.034)	0.71	0.735	2 × 10^–4^	-0.015 (0.035)	0.658	0.705	2 × 10^–4^
1 × 10^–6^	1	0.049 (0.034)	0.157	0.188	0.002	0.037 (0.034)	0.272	0.302	0.001	0.042 (0.035)	0.22	0.254	0.002

Associations for the participants with both genetic and fMRI data available (*N* = 454) can be found in Supplementary Table 2

*PRS PT* p-value threshold of PRS-ADHD, *# of SNPS* number of SNPs used to calculate PRS-ADHD at corresponding p-value threshold, *β* standardized regression coefficients, *SE* standard error, *p-uncor* uncorrected p-value, *p-FDR* FDR-corrected p-value, *R*^*2*^*-PRS* the proportion of variance explained by PRS-ADHD

### PRS-ADHD and behavioral correlates of response inhibition

PRS-ADHD showed significant positive associations with MRT (R^2^-PRS = 0.014; p-FDR = 0.015) and IRT (R^2^-PRS = 0.014; p-FDR = 0.015), but not with SSRT (Table 3). Therefore, MRT and IRT were selected as the behavioral correlates of interest for the subsequent analyses.

**Table 3:** Associations of polygenic risk score for ADHD at the p-value threshold of 1 (PRS-ADHD) with stop-signal task outcomes and task-related neural activity

Dependent variable (*N* = 454)		β (SE)	p-uncor	p-FDR	R^2^-PRS
Stop-signal task outcomes	MRT	0.123 (0.047)	0.01	0.015	0.014
	IRT	0.122 (0.046)	0.008	0.015	0.014
	SSRT	0.053 (0.049)	0.285	0.285	0.003
Task-related neural activity					
Successful inhibition—go	Left fronto-insular regions and putamen	−0.208 (0.047)	1.09 × 10^–5^	1.09 × 10^–5^	0.041
Failed inhibition—go	Left temporal pole and anterior PHG	0.212 (0.047)	7 × 10^–6^	1.17 × 10^–5^	0.042
	Right putamen	0.213 (0.048)	1.06 × 10^–5^	1.33 × 10^–5^	0.044
Failed—successful inhibition	Left fronto-insular, putamen, anterior temporal regions, and PHG	0.273 (0.047)	1.44 × 10^–8^	7.2 × 10^–8^	0.071
	Right basal ganglia and thalamus	0.242 (0.048)	7.23 × 10^–7^	1.81 × 10^–6^	0.056

*MRT* mean reaction time to go-stimuli, *IRT* intra-individual coefficient of variation of reaction time to go-stimuli, *SSRT* stop-signal reaction time, *β* standardized regression coefficients; SE, standard error, *p-uncor* uncorrected p-value, *p-FDR* FDR-corrected p-value, *R*^*2*^*-PRS* the proportion of variance explained by PRS-ADHD, *PHG* parahippocampal gyrus

### PRS-ADHD and neural correlates of response inhibition

#### Successful inhibition—go

**Fig. 1:**
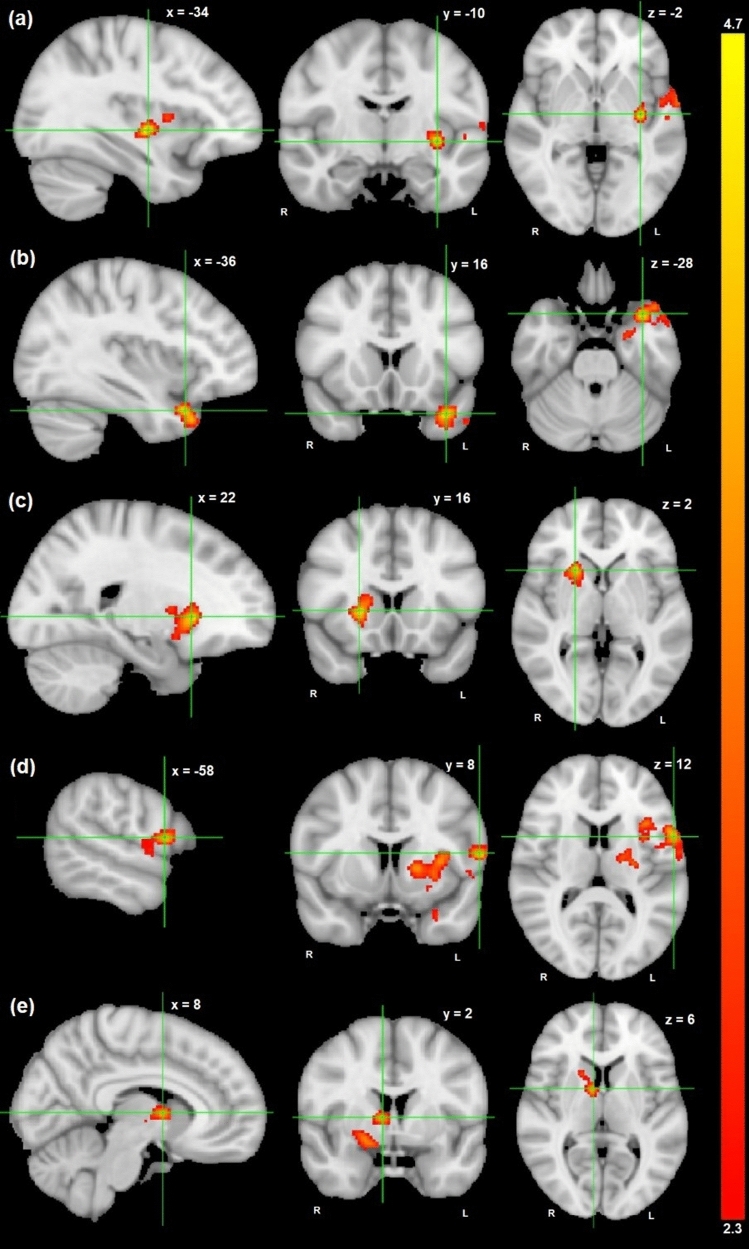
Brain regions that were (**a**) negatively correlated with PRS-ADHD at *x* = −34, *y* = −10, *z* = −2 during successful inhibition—go, (b-c) positively correlated with PRS-ADHD at *x* = −36, *y* = 16, *z* = −28 (**b**) and at *x* = 22, *y* = 16, *z* = 2 (**c**) during failed inhibition—go, and (d-e) positively correlated with PRS-ADHD at *x* = −58, *y* = 8, *z* = 12 (**d**) and at *x* = 8, *y* = 2, *z* = 6 (**e**) during failed—successful inhibition, shown in radiologic view with the right brain shown on the left. The color bar represents Z-scores (2.3–4.7)

For successful inhibition—go contrast, there was a significant negative association between PRS-ADHD and activation in the left fronto-insular regions and putamen (Z = 4.7, p-FWER = 6.25 × 10^–4^, 855 voxels) (Supplementary Table 3, Fig. 1a). Post hoc analyses revealed that 4.1% of the variance in the cluster-average activity was explained by PRS-ADHD (Table 3).

### Failed inhibition—go

For failed inhibition—go contrast, there was significant positive associations between PRS-ADHD and activation in the left temporal pole and anterior parahippocampal gyrus (PHG) (*Z* = 4.28, p-FWER = 0.027, 438 voxels) and in the right putamen (*Z* = 4.38, p-FWER = 0.03, 428 voxels) (Supplementary Table 3, Fig. 1b-c). Post hoc analyses showed that PRS-ADHD explained, respectively, 4.2% and 4.4% of the variance in the average activity in these clusters located on the left and right hemisphere (Table 3).

### Failed—successful inhibition

For failed—successful inhibition contrast, there were significant positive associations between PRS-ADHD and activation in the left fronto-insular, putamen, anterior temporal regions, and anterior PHG (*Z* = 4.15, p-FWER = 1.79 × 10^–7^, 2033 voxels) and in the right basal ganglia and thalamus (*Z* = 3.81, p-FWER = 0.01, 609 voxels) (Supplementary Table 3, Fig. 1d–e). Post hoc analyses revealed that PRS-ADHD explained, respectively, 7.1% and 5.6% of the variance in the average activity in these clusters located on the left and right hemisphere (Table 3).

### Behavioral correlates of response inhibition and ADHD symptoms, controlling for PRS-ADHD

MRT was positively associated with total ADHD (p-FDR = 0.002), inattention (p-FDR = 0.01), and hyperactivity-impulsivity symptom scores (p-FDR = 0.003). Likewise, IRT was positively associated with total ADHD (p-FDR = 1.12 × 10^–7^), inattention (p-FDR = 8.08 × 10^–6^), and hyperactivity-impulsivity symptom scores (p-FDR = 2.05 × 10^–7^), adjusting for PRS-ADHD. Details regarding the results are provided in Supplementary Information and Supplementary Table 4. For completeness, the results of the regression analyses between behavioral correlates and ADHD symptoms without controlling for PRS-ADHD are provided in the Supplementary Information and Supplementary Table 6.

### Neural correlates of response inhibition and ADHD symptoms, controlling for PRS-ADHD

The activation in the left temporal pole and anterior PHG was negatively associated with hyperactivity-impulsivity symptom scores (p-FDR = 0.075). This result failed to survive FDR-correction, but was carried forward to the mediation analyses because it showed at least a nominal significant association (*p* = 0.04) with ADHD symptom scores, when adjusting for PRS-ADHD. The cluster-average activity and PRS-ADHD explained, respectively, 1% and 5.2% of the variance in hyperactivity-impulsivity symptom scores. More detailed results can be found in Supplementary Table 5. For completeness, the results of the regression analyses between neural correlates and ADHD symptoms without controlling for PRS-ADHD are provided in Supplementary Information and Supplementary Table 7.

### Mediation analyses

MRT and IRT were the only behavioral correlates associated with both PRS-ADHD and all ADHD symptoms, and we applied FDR correction for a total of six tests [2 mediators (MRT and IRT) × 3 symptom scales (total, inattention and hyperactivity-impulsivity symptoms)]. Regarding neural correlates, only the left temporal pole and anterior parahippocampal gyrus activation during failed inhibition—go contrast was nominally significantly (*p* = 0.04) associated with symptoms of hyperactivity-impulsivity, so there was only one mediation model involving neural correlates and therefore no additional multiple testing correction could be applied here (see also under Methods).

#### Behavioral mediators

**Fig. 2:**
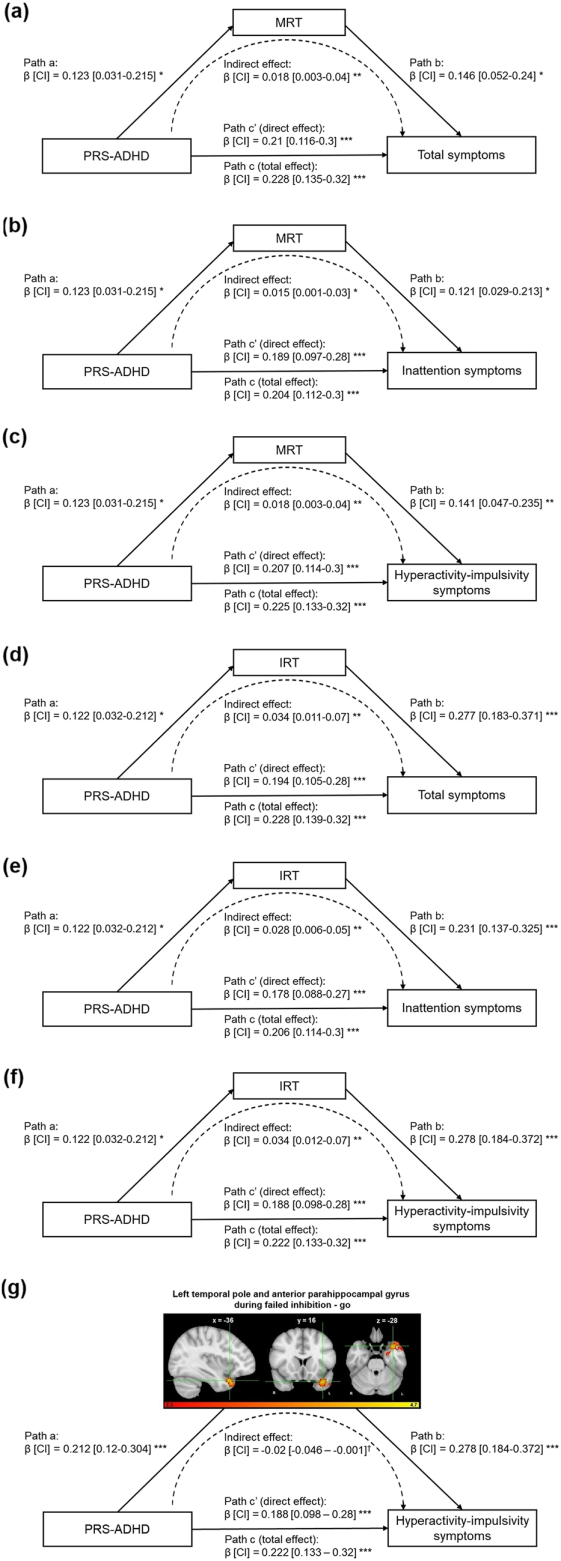
Path diagrams (including standardized regression coefficients and 95% confidence intervals) of the mediation analyses demonstrating that the associations between polygenic risk score for ADHD at *p*-value threshold of 1 (PRS-ADHD) and total ADHD (**a**, **d**), inattention (**b**, **e**), and hyperactivity-impulsivity symptom scores (**c**, **f**, **g**) are mediated by mean reaction time (MRT) (**a**–**c**), intra-individual coefficient of variation of reaction time (IRT) (**d**–**f**), and cluster-average activity in the left temporal pole and anterior parahippocampal gyrus during failed inhibition (**g**). Path “a” represents the effect of PRS-ADHD on the mediator. Path “b” represents the impact of the mediator on ADHD symptom scores controlling for the PRS-ADHD effect. Together, Path “a” and Path “b” represent the indirect (mediated) effect of PRS-ADHD on ADHD symptom scores through the mediator. Path “c” represents the direct effect of PRS-ADHD on ADHD symptom scores and is calculated controlling for the indirect, mediated effect. Path “c” represents the total (mediated and direct) effect of PRS-ADHD on ADHD symptom scores. The asterisks indicate significance using FDR-correction († p-uncorrected < 0.05, * p-FDR < 0.05, ** p-FDR < 0.01, *** p-FDR < 0.001). See also Supplementary Tables 4–5. β, standardized regression coefficients; CI, 95% confidence intervals

As MRT and IRT were associated with ADHD symptoms, controlling for PRS-ADHD (see above), they were selected as potential behavioral mediators. Mediation analyses showed that both MRT and IRT partially mediated the association between PRS-ADHD and ADHD symptoms. Specifically, the association between PRS-ADHD and the total ADHD symptom score was mediated by MRT (indirect effect β = 0.018, 95% CI = (0.003, 0.04); p-FDR = 0.014, accounting for 7.7% of the total effect) and IRT (indirect effect β = 0.034, 95% CI = (0.011, 0.07); p-FDR = 0.012, accounting for 15.7% of the total effect). Similar results were also obtained for the inattention and hyperactivity-impulsivity symptom scores (see Supplementary Information). Figure 2 represents path diagrams of mediation analyses.

#### Neural mediators

As the cluster-average activity in the left anterior temporal pole and PHG during failed inhibition was (nominally significantly) associated with hyperactivity-impulsivity symptoms, controlling for PRS-ADHD (see above), it was selected as potential neural mediator. The association between PRS-ADHD and hyperactivity-impulsivity symptoms was partially mediated by activity in the left temporal pole and anterior PHG during failed inhibition—go [indirect effect β =  −0.02, 95% CI = (−0.046, −0.001); *p* = 0.04, accounting for 9.5% of the total effect). Figure 2 represents path diagrams of mediation analyses.

## Discussion

This study investigated the relationships between genetic liability to ADHD (PRS-ADHD), its core symptoms, and behavioral and functional neural correlates of response inhibition in a sample of children, adolescents, and young adults with ADHD, their unaffected siblings, and healthy controls. A higher genetic liability to ADHD was associated with higher levels of symptom severity in both symptom domains of inattention and hyperactivity-impulsivity, as well as with total ADHD symptom severity. Further, PRS-ADHD were found to be associated with slower and more variable responses to go-stimuli in the stop-signal task and with altered neural activity in several regions of the bilateral fronto-striatal network during response inhibition. We identified behavioral performance in the stop-signal task (MRT and IRT) as partial mediators of the association between PRS-ADHD and ADHD symptoms in both symptom domains; activity in the left temporal pole and anterior parahippocampal gyrus (PHG) during failed inhibition was observed to be a mediator in the relationship of PRS-ADHD with hyperactivity-impulsivity symptoms.

Our finding that PRS-ADHD were positively associated with not only total ADHD symptom scores, but also with inattention and hyperactivity-impulsivity symptom scores, is consistent with our hypothesis and previous studies [26, 50, 51]. The explained variance by PRS-ADHD for both ADHD symptom domains was similar (3.9% for inattention and 4% for hyperactivity-impulsivity).

The significant positive associations that we found between the PRS-ADHD and latency of go responses, as indexed by MRT, and intra-individual reaction time variability, as indexed by IRT, point to an overlap between genetic effects on ADHD and MRT and IRT; it also further supports the hypothesis of increased intra-individual response variability as an endophenotype of ADHD [52]. Elevated reaction time and greater reaction time variability in a cognitive task are among the most consistent findings in the literature of childhood ADHD [53–55] and have been repeatedly observed in stop-signal task studies [14, 55–58]. The unaffected siblings of individuals with ADHD have been shown to have levels of IRT intermediate between probands with ADHD and controls [14]. Further to that, multivariate genetic analyses of ADHD cases and unaffected sibling pairs showed that cognitive impairment in ADHD related to response time (variability) during a Go/No-Go task [59], and a link between PRS-ADHD and reaction time variability in response inhibition tasks have also been recently reported [29, 60].

There was no significant association of PRS-ADHD with SSRT, the core measure of inhibitory control during the stop-signal task. Impaired response inhibition, as indexed by greater SSRT values, is thought to be one of the primary deficits associated with ADHD [58, 61, 62]. Nevertheless, previous stop-signal task studies (sample sizes ranging from 45 to 170) indeed reported shorter, but also *similar* SSRT values in children with ADHD compared to healthy controls [63–66]. Moreover, our results are in line with a recent study that reported no association between PRS-ADHD and inhibitory control, as indexed by commission errors during a Go/No-Go task [29] and SSRT during stop-signal tasks [60, 67]. However, a link between PRS-ADHD and cognitive interference, measured in the Stroop task, has also been reported [28]. These somewhat inconsistent results in the current literature might result from different samples (the previous studies were limited to individuals with ADHD [28, 29], whereas we also included unaffected siblings and healthy controls) and/or different experimental tasks (since each inhibitory paradigm has its own measure of inhibitory control). As suggested earlier [29, 60, 67], the genetic variants captured by the PRS-ADHD might not be directly related to the core behavioral measures of response inhibition, but rather to other response inhibition-related components (MRT, IRT) and neural activity. It is also possible that shared genetic effects between ADHD and certain behavioral correlates of response inhibition (i.e., SSRT) are more subtle than what we can detect with our current ‘base’ and ‘target’ samples. Therefore, larger future studies are needed to more robustly confirm genetic sharing of ADHD with different behavioral performance measures of response inhibition across different experimental paradigms. All in all, our findings point to the influence of genetic liability to ADHD on attentional processing during response inhibition rather than inhibitory control as such.

To our knowledge, this is the first study to investigate a possible shared genetic background between ADHD and brain activation in the response inhibition network by using individual-level PRS and fMRI data. PRS-ADHD was significantly related to activity of several regions in the bilateral fronto-striatal-thalamo-cortical network associated with response inhibition. We identified a cluster within the left fronto-insular regions and putamen, for which activity during successful inhibition was negatively associated with the PRS-ADHD. To further investigate the brain activation related to failed response inhibition, we used two separate contrasts, “failed inhibition—go” and “failed—successful inhibition”, which provide complementary information about the neural mechanisms underlying response inhibition (the first contrast is thought to reflect such as the engagement of the inhibitory control network and the detection of a stop signal, while the second contrast compares error processing and adjustment) [68, 69]. The failed inhibition—go contrast revealed two clusters positively associated with PRS-ADHD, located in the left temporal pole and anterior PHG, and in the right putamen. In failed—successful inhibition contrasts, positive associations of PRS-ADHD with neural activation were found within two clusters localized in the left fronto-insular, putamen, anterior temporal, and parahippocampal regions, and in the right thalamus and basal ganglia.

Our findings regarding neural activity converge with a previous meta-analysis (607 participants; 287 ADHD cases and 320 healthy controls), which reported aberrant activation in individuals with ADHD during response inhibition for a large neural network encompassing these same areas [12]. Moreover, decreased activation in bilateral fronto-parietal and fronto-striatal regions during the stop-signal task has also been reported in unaffected siblings of individuals with ADHD when compared to healthy controls in a previous study that also used the NeuroIMAGE sample (420 participants; 185 ADHD cases, 111 of their unaffected siblings, and 124 healthy controls) [14]. During failed inhibition, we also identified a cluster in the left anterior temporal pole and PHG, in addition to areas of inhibition in the basal ganglia whose activities were positively associated with PRS-ADHD. The PHG with its surrounding areas, such as the entorhinal cortex and hippocampus, has been associated with post-error processing and error-driven learning strategy [70]. The positive association between PRS-ADHD and activity in the left PHG may therefore reflect different strategies adopted by the individuals with higher PRS-ADHD for performance-monitoring and error-processing during the stop-signal task. These results, combined with our findings regarding the significant associations of PRS-ADHD with the activity of key nodes in the response inhibition network such as the prefrontal areas, anterior cingulate cortex, basal ganglia, and thalamus, suggest that common risk variants for ADHD play a role in altered neural substrates of inhibitory control in ADHD.

Our finding that MRT and IRT mediated the association between PRS-ADHD and the total ADHD, inattention, and hyperactivity-impulsivity symptom scores confirms disrupted attentional processing during response inhibition as a key cognitive variable in the context of ADHD. Individuals who had a greater polygenic risk for ADHD showed slower go responses with greater variability in response time, which in turn partially mediated the link between PRS-ADHD and ADHD symptom severity. MRT and IRT varied with regard to how much of the total effect they mediated. MRT mediated 7.7% of the total association of PRS-ADHD with total ADHD symptom severity, whereas the corresponding percentage for IRT was 15.7%. Similar patterns emerged for the inattention and hyperactivity-impulsivity symptom scores. A recent study also demonstrated that reaction time variability is associated with PRS-ADHD and also partially mediated the relationship between PRS-ADHD and ADHD traits [60]. Furthermore, increased reaction time variability has repeatedly been reported in children and adults with ADHD [54, 71–73]. Moreover, reaction time variability in particular has been suggested to be a robust and reliable feature of ADHD across stop-signal and other cognitive tasks [71]. Thus, higher genetic liability to ADHD, as indexed by higher PRS-ADHD, might lead to the development of more ADHD symptoms somewhat more clearly through altered IRT than MRT as a possible intermediate phenotype.

As for the neural correlates of response inhibition, the association of PRS-ADHD with the severity of hyperactivity-impulsivity symptoms was partially mediated by lower activity in the left temporal pole and anterior PHG during failed inhibition. As the indirect effect of the neural activation was negative while the total effect had a positive sign, the effect of neural activation as a mediator indirectly reduced the effect of PRS-ADHD on ADHD symptoms, which may suggest a ‘suppressive’ or inhibiting (neural mediation) effect. More specifically, a subset of the SNPs that drive the direct effect of PRS-ADHD on the severity of hyperactivity-impulsivity symptoms might also be involved in the above-mentioned indirect pathway involving activation in the left temporal pole and anterior PHG during failed inhibition. Given the association between the PHG with its surrounding areas and post-error processing [70], the increased activation of the anterior temporal pole and PHG in participants with a high genetic liability to ADHD may represent a compensatory response to error processing. It can be speculated that these individuals leverage their errors during failed inhibition to help optimize future behavior in upcoming trials in the stop-signal task and might develop a strategy to develop better general behavioral control skills to self-regulate their non-optimal impulsive behavior. However, because the association between the neural activity in the left temporal pole and anterior PHG and the hyperactivity-impulsivity symptoms was only nominally significant, further research is warranted.

Our results should be interpreted in light of the study’s potential strengths and limitations. As a unique feature, we consider the inclusion of individuals with ADHD, their unaffected siblings, and controls in our ‘target’ sample, which together may represent a more comprehensive and representative range of ADHD symptomatology and response inhibition correlates than a case-only study. Our PRS-ADHD—based on a well-powered GWAS of ADHD—showed a robust association with (both inattention and hyperactivity-impulsivity) ADHD symptoms in our ‘target’ sample. Nevertheless, a possible limitation of our current study could be the somewhat modest sample size of NeuroIMAGE. Future studies would benefit from larger ‘target’ sample sizes and probably even more powerful GWAS to allow for more definite conclusions about the shared genetic architecture between ADHD and behavioral and neural correlates of response inhibition. It is also worth keeping in mind that fMRI detects the hemodynamic changes in cerebral blood flow rather than direct neural activity, and our findings of fMRI analysis reflect altered brain activation related to cognitive processing during a stop-signal task. The clinical interpretation of altered brain activity (i.e., whether findings may reflect pathological or just altered physiological brain functioning) remains complicated. Although we used cross-sectional rather than longitudinal data to investigate mediation effects, the use of genetic risk scores together with the nature of brain functioning and (neurodevelopmental) behavioral symptomatology means that in this case inference of causality with regard to temporal precedence is not necessarily limited by the study design.

To conclude, our findings provide evidence for and better understanding of a shared genetic etiology between ADHD and behavioral measures and neural activity related to response inhibition in youth with a diagnosis of ADHD, unaffected siblings, and controls, corroborating response inhibition as a potential endophenotype. Partial mediation effect of brain activation in the left temporal pole and anterior PHG during failed inhibition on the association of PRS-ADHD with severity of hyperactivity-impulsivity symptoms may point to a possible pathway from genetic liability for ADHD to the expression of hyperactivity-impulsivity symptoms through altered brain activation during response inhibition. Moreover, MRT and IRT partially mediated the relationships of PRS-ADHD and ADHD symptom severity, suggesting that genetic liability to ADHD influences attention regulation, which in turn may affect the severity of both inattention and hyperactivity-impulsivity symptoms. Overall, our findings lend support for the conceptualization of response inhibition as a neurobiological mechanism underlying the etiology of ADHD. Our findings also provide novel insights regarding the genetic sharing of ADHD symptomatology with cognitive and underlying neural processing related to response inhibition.

### Electronic supplementary material

Below is the link to the electronic supplementary material.

Supplementary file1 (DOCX 198 kb)
