# Supplementary material for: Shared genetic etiology between ADHD, task-related behavioral measures and brain activation during response inhibition in a youth ADHD case–control study
Source: Eur Arch Psychiatry Clin Neurosci. 2023 Jun 28;274(1):45–58. doi: 10.1007/s00406-023-01632-8 (PMC10786981; doi:10.1007/s00406-023-01632-8)
Supplement: Supplementary file 1 — Supplementary file1 (DOCX 198 kb) [file 406_2023_1632_MOESM1_ESM.docx]

**SUPPLEMENTARY INFORMATION**

**METHODS**

**Data and participants**

The data used in this study is a part of the NeuroIMAGE project, which is a Dutch follow-up of the International Multicenter ADHD Genetics (IMAGE) project. NeuroIMAGE includes neurocognitive, neuroimaging, and genetic data as well as environmental factors of individuals with ADHD, their unaffected siblings, and healthy controls [1]. The genetic and imaging data used in this study were collected at two centers: Vrije Universiteit Amsterdam (Amsterdam, the Netherlands) and Radboud University Medical Center (Nijmegen, the Netherlands).

General exclusion criteria for all participants were not being of European Caucasian descent, an IQ < 70, contraindications for fMRI, a diagnosis of autism spectrum disorder, epilepsy, general learning difficulties, brain disorders, or any known genetic disorder.

**Genotyping and data preprocessing**

DNA was extracted from whole-blood or saliva and all participants were genotyped using the Illumina PsychChip, in two batches (N=192 and N=798). Batches were merged in Plink 1.9 (https://www.cog-genomics.org/plink/1.9/) [2, 3]. Sex chromosomes, insertions, deletions, other non-SNP variation; and duplicate, ambiguous and mismatching SNPs between batches were excluded. Genome-wide data was further processed using RICOPILI (Rapid Imputation for COnsortias PIpeLIne for GWAS) pipeline [4]. Exclusion criteria for SNPs were call rate <0.05, deviation from Hardy-Weinberg equilibrium P<10^-6^, minor allele frequency <0.01. SNPs were also excluded if the missing-rate difference between batches was >0.01. Exclusion criteria for individuals were: call rate <0.05, inconsistent sex between self-report and GWAS data, identity by descent with another participant (quasi-random exclusion for each pair, with priority to more complete phenotyping data). After principal component analysis (PCA) in RICOPILI, 18 individuals were excluded for deviating from the main cluster of the sample defined by any of the first 4 principal components (>3 standard deviations). None of the principal components (PCs) were associated with genotyping batch. 48 SNPs were excluded for a batch-effect of P<0.0008 (this threshold was determined by deviation from the diagonal in the QQ-plot of genome-wide batch-effects). After all quality control steps, 283,843 SNPs and 952 individuals were retained, and the PCA step was re-run on this sample to obtain new PCs for subsequent analyses. Imputation was run in RICOPILI using minimac3 and Phase3 of the European 1000 genomes as reference. The strict best-guess output from RICOPILI (genotype call P>0.8, SNP missing rate < 0.01, MAF>0.05, 2,840,886 SNPs), was used for the PRS analysis.

**fMRI paradigm: stop-signal task**

A visual version of the stop-signal task was used to probe the behavioral and neural mechanisms of response inhibition [5]. Participants performed a two-choice reaction task in which they were required to respond as quickly as possible to a horizontal left or right pointing arrow (go-signal) by pressing left or right button, respectively, unless it was a stop trial in which the go-signal was followed by an arrow pointing upwards (stop-signal). In 25% of trials (stop trials), the stop-signal was presented after a variable delay after the appearance of the go-signal, the so-called “stop-signal delay”, indicating that participants were required to interrupt the already initiated reaction and to suppress their responses. We used a performance-adjusted stop-signal task in which the initial 250-ms stop-signal delay was continuously adapted to the individual response time of the participant by decreasing or increasing it 50 ms after a successful or unsuccessful stop trial, respectively, so that an approximately 50% success rate on the stop trials for all participants was achieved. The task included two practice and four experimental blocks consisting of 60 trials (48 go- and 12 stop-trials).

**fMRI acquisition**

Data were acquired at two scanning locations on similar 1.5 Tesla Siemens scanners using identical protocols (Siemens Sonata at Vrije Universiteit Amsterdam in Amsterdam, the Netherlands, and Siemens Avanto at Radboud University Medical Center in Nijmegen, the Netherlands). The stop-signal paradigm was embedded in a longer scanning session that included other fMRI and structural scans. The stop-signal fMRI data were collected in four subsequent fMRI runs separated by 1-minute breaks using a T2*weighted echo planar imaging sequence (TR=2400 ms, TE=40 ms, FOV=224 mm, voxel size=3x3x3.5 mm, 94 Volumes per block). For spatial localization and normalization, we included each participant’s high resolution MPRAGE T1 scan (TR=2730ms, TE=2.95ms, voxel size=1x1x1mm, FOV=256mm, 76 slices).

**fMRI preprocessing**

The fMRI data were preprocessed using FSL tools (FMRIB's Software Library, www.fmrib.ox.ac.uk/fsl; fMRI Expert Analysis Tool, version 6.0) [6]. Preprocessing included removal of the first four volumes of each run to allow magnetization equilibration, head motion correction by volume-realignment to the middle volume using MCFLIRT [7], slice-timing correction, global 4D mean intensity normalization, and spatial smoothing using a 6-mm FWHM Gaussian kernel. Additional motion-related artifacts were detected in single-subject data applying the ICA-AROMA protocol [8], which is freely available through GitHub (https://github.com/maartenmennes/ICA-AROMA). Subsequently, mean signals from CSF and white matter were regressed out using subject-level masks obtained by multiplying subject-level CSF and white matter segmentations with the MNI152-based CSF and white matter masks provided as part of FSL. Finally, we applied a 0.01 Hz temporal high-pass filter (Gaussian-weighted least square straight line fit to the data). For each participant, all acquisitions were registered to its high-resolution T1 image using Boundary-Based Registration available in FSL FLIRT^9^. All high-resolution T1 images were registered to MNI152 space using 12-dof linear registration available in FLIRT [7, 9, 10], and further refined using non-linear registration available in FSL FNIRT [11].

**Multiple testing correction using false discovery rate**

A false discovery rate (FDR) [12] was applied to correct for multiple testing.

1) The associations between PRS-ADHD and ADHD symptoms: The FDR was applied to correct for multiple testing (N=30) for PRS-ADHD at all *p-value* thresholds across all three symptom scales (10 PRS-ADHD *p-value* thresholds x 3 symptom scales).

2a) The associations between PRS-ADHD and behavioral correlates of response inhibition: The FDR was applied to correct for multiple testing (N=3) for PRS-ADHD at *p-value* threshold = 1 across all three behavioral outcomes (1 PRS-ADHD *p-value* threshold x 3 behavioral outcomes).

2b) The associations between PRS-ADHD and neural correlates of response inhibition: The FDR was applied to correct for multiple testing (N=5) for PRS-ADHD at *p-value* threshold = 1 across all five clusters found to be significantly associated with PRS-ADHD (1 PRS-ADHD *p-value* threshold x 5 neural clusters).

3a) The associations between behavioral correlates of response inhibition and ADHD symptoms, controlling for PRS-ADHD: The FDR was applied to correct for multiple testing (N=6) for two behavioral outcomes across all three symptom scales (2 behavioral outcomes x 3 symptom scales).

3b) The associations between neural correlates of response inhibition and ADHD symptoms, controlling for PRS-ADHD: The FDR was applied to correct for multiple testing (N=15) for five neural clusters across all three symptom scales (5 neural clusters x 3 symptom scales).

Mediation analyses: FDR correction was applied to control for multiple testing separately for behavioral and neural mediators across symptom scales that were found to be significantly associated with the mediator of interest (see 3a and 3b).

**RESULTS**

**The associations between behavioral correlates of response inhibition and ADHD symptoms, controlling for PRS-ADHD**

MRT explained 1.9%, 1.3%, and 1.6% of the variance in total ADHD, inattention, and hyperactivity-impulsivity symptom scores, respectively, while 4.2%, 3.4%, and 4.1% of the variance in total ADHD, inattention, and hyperactivity-impulsivity symptom scores was explained by PRS-ADHD. IRT explained 6.3%, 4.3%, and 5.9% of the variance in total ADHD, inattention, and hyperactivity-impulsivity symptom scores, respectively, while 3.8%, 3.2%, and 3.5% of the variance in total ADHD, inattention, and hyperactivity-impulsivity symptom scores was explained by PRS-ADHD. Detailed results can be found in Supplementary Table 4.

**Mediation analyses**

*Behavioral mediators*

Mediation analyses showed that both MRT and IRT partially mediated the association of PRS-ADHD with the inattention and hyperactivity-impulsivity symptom scores. The association between PRS-ADHD and respectively inattention and hyperactivity-impulsivity symptoms was mediated by MRT (*inattention*: indirect effect β = 0.015, 95% CI = [0.001, 0.03]; p-FDR = 0.021, accounting for 6.7% of the total effect; *hyperactivity-impulsivity*: indirect effect β = 0.018, 95% CI = [0.003, 0.04]; p-FDR = 0.014, accounting for 7.9% of total effect) and IRT (*inattention*: indirect effect β = 0.028, 95% CI = [0.006, 0.05]; p-FDR = 0.014, accounting for 13.3% of the total effect; *hyperactivity-impulsivity*: indirect effect β = 0.034, 95% CI = [0.012, 0.07]; p-FDR = 0.012, accounting for 16.5% of the total effect).

**Additional analyses**

**Associations between behavioral correlates of response inhibition and ADHD symptoms**

Mixed model regression analyses (using R package lme4 [13]) were performed to test for associations between behavioral outcomes of the stop-signal tasks, namely MRT, IRT, and SSRT, and ADHD symptoms. Family identity was included as a random variable, and age, sex, and fMRI scanning site were entered as covariates. The FDR was applied to correct for multiple testing (N=9) for three behavioral outcomes across all three symptom scales (3 behavioral outcomes x 3 symptom scales), and the results that survived after this correction at p-FDR < 0.05 were considered as significant. A summary of the results can be found in Supplementary Table 6.

MRT was significantly positively associated with total ADHD (β = 0.182, 95% CI = [0.088, 0.276]; p-uncorrected = 1.84x10^-4^, p-FDR = 4.14x10^-4^), inattention (β = 0.154, 95% CI = [0.062, 0.246]; p-uncorrected = 0.001, p-FDR = 1.5x10^-3^), and hyperactivity-impulsivity symptom score (β = 0.176, 95% CI = [0.084, 0.273]; p-uncorrected = 2.62x10^-4^, p-FDR = 4.72x10^-4^). IRT was also significantly positively associated with total ADHD (β = 0.307, 95% CI = [0.211, 0.403]; p-uncorrected = 1.02x10^-9^, p-FDR = 4.59x10^-9^), inattention (β = 0.257, 95% CI = [0.163, 0.351]; p-uncorrected = 1.92x10^-7^, p-FDR = 5.76x10^-7^), and hyperactivity-impulsivity symptom scores (β = 0.311, 95% CI = [0.215, 0.407]; p-uncorrected = 7.04x10^-10^, p-FDR = 4.59x10^-9^). SSRT was only significantly positively associated with inattention symptom scores (β = 0.118, 95% CI = [0.028, 0.208]; p-uncorrected = 0.011, p-FDR = 0.014).

**Associations between neural correlates of response inhibition and ADHD symptoms**

Mixed model regression analyses (using R package lme4 [13]) were performed to test for associations between neural activation during the contrasts of interest and ADHD symptoms. Family identity was included as a random variable, and age, sex, and fMRI scanning site were entered as covariates. The FDR was applied to correct for multiple testing (N=15) for five clusters found to be significantly associated with PRS-ADHD across all three symptom scales (5 neural clusters x 3 symptom scales). As and the results that survived after this correction at p-FDR < 0.05 were considered as significant. A summary of the results can be found in Supplementary Table 7.

The activation in the right putamen during failed inhibition – go was associated with inattention symptom scores (β = 0.105, 95% CI = [0.017, 0.193]; p-uncorrected = 0.021, p-FDR = 0.205). The activation in the right basal ganglia and thalamus during failed – successful inhibition was associated with total ADHD (β = 0.095, 95% CI = [0.005, 0.185]; p-uncorrected = 0.041, p-FDR = 0.205), and inattention symptom scores (β = 0.105, 95% CI = [0.008, 0.184]; p-uncorrected = 0.034, p-FDR = 0.205). However, these results failed to survive FDR correction.

**REFERENCES**

1. von Rhein D, Mennes M, van Ewijk H, et al (2015) The NeuroIMAGE study: a prospective phenotypic, cognitive, genetic and MRI study in children with attention-deficit/hyperactivity disorder. Design and descriptives. Eur Child Adolesc Psychiatry 24:. https://doi.org/10.1007/s00787-014-0573-4

2. Purcell S, Neale B, Todd-Brown K, et al (2007) PLINK: A tool set for whole-genome association and population-based linkage analyses. Am J Hum Genet 81:. https://doi.org/10.1086/519795

3. Chang CC, Chow CC, Tellier LCAM, et al (2015) Second-generation PLINK: Rising to the challenge of larger and richer datasets. Gigascience 4:. https://doi.org/10.1186/s13742-015-0047-8

4. Lam M, Awasthi S, Watson HJ, et al (2020) RICOPILI: Rapid Imputation for COnsortias PIpeLIne. Bioinformatics 36:. https://doi.org/10.1093/bioinformatics/btz633

5. Logan GD, Cowan WB, Davis KA (1984) On the ability to inhibit simple and choice reaction time responses: A model and a method. J Exp Psychol Hum Percept Perform 10:. https://doi.org/10.1037/0096-1523.10.2.276

6. Jenkinson M, Beckmann CF, Behrens TEJ, et al (2012) FSL. Neuroimage 62:782–790. https://doi.org/https://doi.org/10.1016/j.neuroimage.2011.09.015

7. Jenkinson M, Bannister P, Brady M, Smith S (2002) Improved optimization for the robust and accurate linear registration and motion correction of brain images. Neuroimage 17:. https://doi.org/10.1016/S1053-8119(02)91132-8

8. Pruim RHR, Mennes M, van Rooij D, et al (2015) ICA-AROMA: A robust ICA-based strategy for removing motion artifacts from fMRI data. Neuroimage 112:. https://doi.org/10.1016/j.neuroimage.2015.02.064

9. Greve DN, Fischl B (2009) Accurate and robust brain image alignment using boundary-based registration. Neuroimage 48:. https://doi.org/10.1016/j.neuroimage.2009.06.060

10. Jenkinson M, Smith S (2001) A global optimisation method for robust affine registration of brain images. Med Image Anal 5:. https://doi.org/10.1016/S1361-8415(01)00036-6

11. Anderson J, Jenkinson M, Smith S (2007) Non-linear registration aka Spatial normalisation. FMRIB Technical Report TR07JA2

12. Benjamini Y, Hochberg Y (1995) Controlling the False Discovery Rate: A Practical and Powerful Approach to Multiple Testing. J R Stat Soc Ser B 57:289–300. https://doi.org/https://doi.org/10.1111/j.2517-6161.1995.tb02031.x

13. Bates D, Mächler M, Bolker BM, Walker SC (2015) Fitting linear mixed-effects models using lme4. J Stat Softw 67:. https://doi.org/10.18637/jss.v067.i01

14. Wechsler D (2003) Wechsler intelligence scale for children, fourth edition

15. Keith Conners C, Sitarenios G, Parker JDA, Epstein JN (1998) The revised Conners’ Parent Rating Scale (CPRS-R): Factor structure, reliability, and criterion validity. J Abnorm Child Psychol 26:. https://doi.org/10.1023/A:1022602400621


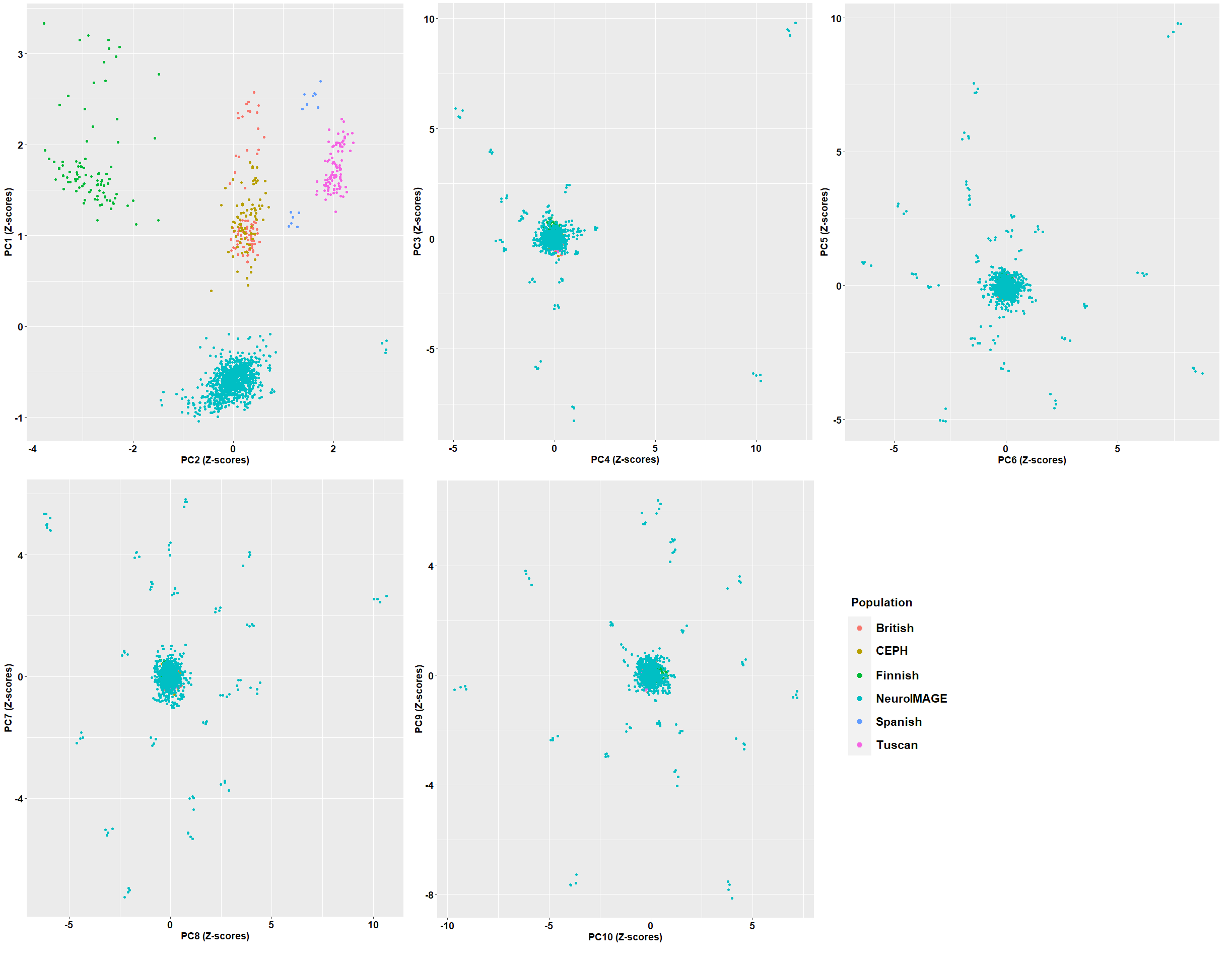


Supplementary Fig. 1. Scatterplots of the first ten principal components colored by 1KG European populations (British, CEPH, Finnish, Spanish, and Tuscan) and NeuroIMAGE sample. The NeuroIMAGE clusters closely with European British and CEPH populations, while showing some variation towards Finnish, Spanish, and Tuscan populations when looking at the first two principal components.


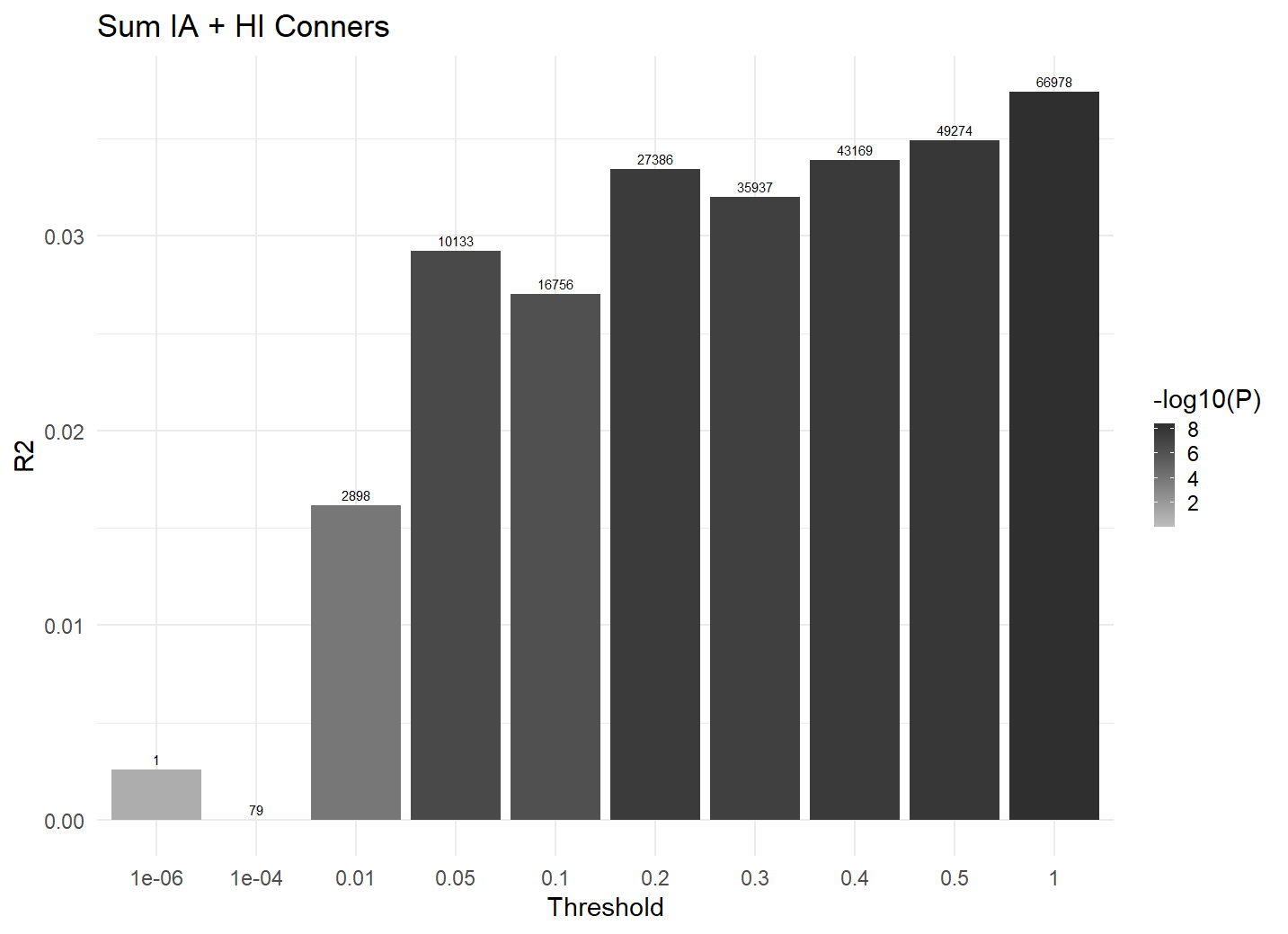


Supplementary Fig. 2. Bar plot showing at broad *p-value* thresholds for PRS-ADHD predicting total ADHD symptom scores in all participants with genetic data available (N=845). Each bar represents the respective p-value thresholds whereas the numbers above bars denote the number of SNPs used to calculate PRS-ADHD at that threshold.


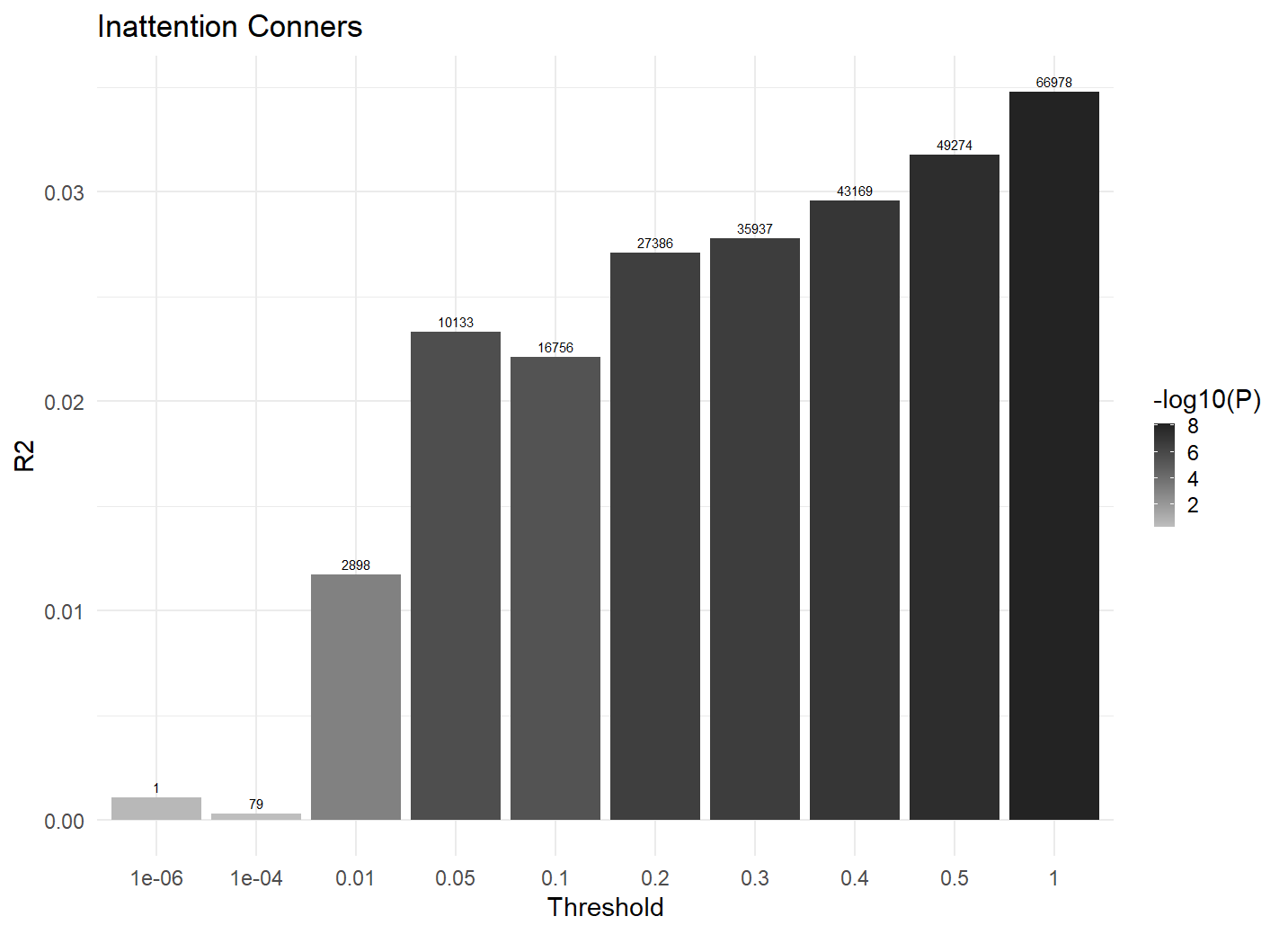


Supplementary Fig. 3*.* Bar plot showing at broad *p-value* thresholds for PRS-ADHD predicting inattention symptom scores in all participants with genetic data available (N=859). Each bar represents the respective p-value thresholds whereas the numbers above bars denote the number of SNPs used to calculate PRS-ADHD at that threshold.


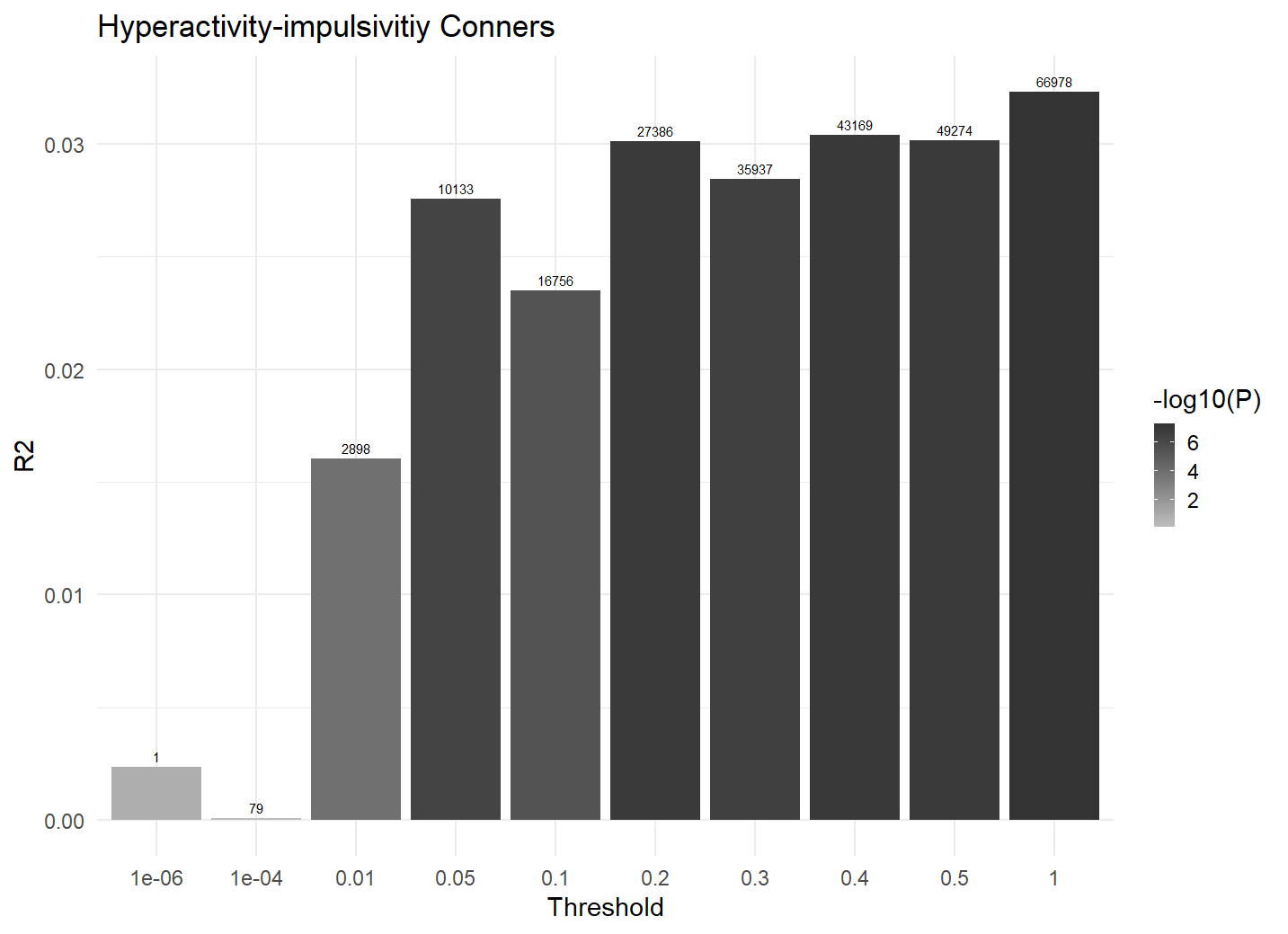


Supplementary Fig. 4. Bar plot showing at broad *p-value* thresholds for PRS-ADHD predicting hyperactivity-impulsivity symptom scores in all participants with genetic data available (N=866). Each bar represents the respective p-value thresholds whereas the numbers above bars denote the number of SNPs used to calculate PRS-ADHD at that threshold.

Supplementary Table 1 Demographic characteristics of all participants with genetic data available.

| *Demographic characteristics* | | | | |
| --- | --- | --- | --- | --- |
|  | N |  | | |
| Sex (female/male) | 908 | 394 (43.4%)/ 514 (56.6%) | | |
| Medication use (yes/no) | 903 | 161 (17.8%)/ 742 (82.2%) | | |
| Handedness (right/left) | 814 | 98 (12%)/ 716 (88%) | | |
|  | N | Mean | SD | Range |
| Age | 908 | 16.92 | 3.69 | [5.77 – 30.51] |
| Estimated IQ^a^ | 814 | 99.69 | 16.07 | [46 – 147] |
| Total symptom score^b^ | 844 | 12.95 | 12.54 | [0 – 53] |
| Inattention symptom score^b^ | 865 | 8.11 | 7.39 | [0 – 27] |
| Hyperactivity-impulsivity symptom score^b^ | 858 | 5.08 | 5.98 | [0 – 27] |

N, number of participants with data available; total symptom score, sum of the scores on the cognitive problems/inattention and hyperactivity subscales of the Conners’ Parent Rating Scale - Revised: Long version (CPRS-R:L); inattention symptom score, scores on the cognitive problems/inattention subscale of the CPRS-R:L; hyperactivity-impulsivity symptom score, scores on the hyperactivity subscale of the CPRS-R:L.

^a^ Based on the block-design and vocabulary subtests of the Wechsler Intelligence Scale for Children or Wechsler Adult Intelligence Scale [14]

^b^ Scores on the Conners’ Parent Rating Scale - Revised: Long version [15]

Supplementary Table 2 Associations between polygenic risk score for ADHD (PRS-ADHD) at a range of p-value thresholds and total, inattention, and hyperactivity-impulsivity symptom scores for the participants with both genetic and fMRI data available.

| Independent  variable | | Dependent variable | | | | | | | | | | | |
| --- | --- | --- | --- | --- | --- | --- | --- | --- | --- | --- | --- | --- | --- |
|  |  | Total symptom score (N=423) | | | | Inattention symptoms (N=438) | | | | Hyperactivity-impulsivity symptoms (N=431) | | | |
| PRS PT | # of SNPs | β (SE) | p-uncor | p-FDR | R^2^-PRS | β (SE) | p-uncor | p-FDR | R^2^-PRS | β (SE) | p-uncor | p-FDR | R^2^-PRS |
| 1 | 66,978 | 0.23 (0.047) | 1.74x10^-6^ | 1.31x10^-5^ | 0.051 | 0.204 (0.046) | 1.46x10^-5^ | 2.92x10^-5^ | 0.04 | 0.229 (0.048) | 2.22x10^-6^ | 9.51x10^-6^ | 0.05 |
| 0.5 | 49,274 | 0.22 (0.048) | 5.1x10^-6^ | 1.53x10^-5^ | 0.047 | 0.191 (0.047) | 4.91x10^-5^ | 7.75x10^-5^ | 0.036 | 0.224 (0.048) | 4.07x10^-6^ | 1.36x10^-5^ | 0.048 |
| 0.4 | 43,169 | 0.223 (0.047) | 3.83x10^-6^ | 1.47x10^-5^ | 0.048 | 0.192 (0.047) | 4.47x10^-5^ | 7.45x10^-5^ | 0.036 | 0.23 (0.048) | 2.14x10^-6^ | 1.07x10^-5^ | 0.051 |
| 0.3 | 35,937 | 0.23 (0.048) | 1.96x10^-6^ | 1.18x10^-5^ | 0.051 | 0.202 (0.047) | 1.92x10^-5^ | 3.6x10^-5^ | 0.039 | 0.234 (0.048) | 1.69x10^-6^ | 1.69x10^-5^ | 0.052 |
| 0.2 | 27,386 | 0.243 (0.047) | 4.67x10^-7^ | 7x10^-6^ | 0.057 | 0.205 (0.047) | 1.39x10^-5^ | 2.98x10^-5^ | 0.041 | 0.251 (0.048) | 2.35x10^-7^ | 7.05x10^-6^ | 0.06 |
| 0.1 | 16,756 | 0.212 (0.048) | 1.2x10^-5^ | 3x10^-5^ | 0.044 | 0.179 (0.047) | 1.59x10^-4^ | 2.38x10^-4^ | 0.032 | 0.218 (0.048) | 8.49x10^-6^ | 2.32x10^-5^ | 0.046 |
| 0.05 | 10,133 | 0.206 (0.048) | 2.51x10^-5^ | 4.43x10^-5^ | 0.042 | 0.176 (0.047) | 2.32x10^-4^ | 3.31x10^-4^ | 0.032 | 0.214 (0.049) | 1.3x10^-5^ | 2.99x10^-5^ | 0.045 |
| 0.01 | 2,898 | 0.122 (0.049) | 0.013 | 0.017 | 0.016 | 0.084 (0.048) | 0.08 | 0.1 | 0.008 | 0.142 (0.049) | 0.004 | 0.005 | 0.02 |
| 1x10^-4^ | 79 | 0.009 (0.049) | 0.86 | 0.86 | 1x10^-4^ | 0.02 (0.048) | 0.674 | 0.749 | 4x10^-4^ | 0.024 (0.049) | 0.631 | 0.728 | 7x10^-4^ |
| 1x10^-6^ | 1 | -0.014 (0.048) | 0.777 | 0.804 | 3x10^-4^ | -0.016 (0.047) | 0.733 | 0.785 | 3x10^-4^ | -0.026 (0.049) | 0.586 | 0.703 | 8x10^-4^ |

N, number of participants with data available; PRS PT, p-value threshold of PRS-ADHD; # of SNPS, number of SNPs used to calculate PRS-ADHD at corresponding p-value threshold; β, standardized regression coefficients; SE, standard error; p-uncor, uncorrected p-value; p-FDR, FDR-corrected p-value; R^2^-PRS, the proportion of variance explained by PRS-ADHD.

Supplementary Table 3 Location, maximum z-value, size and p-value of the clusters correlating with PRS-ADHD.

| Association with PRS-ADHD | x, y, z (MNI) | Z-value | Cluster size (in voxels) | p-FWER | Brain regions |
| --- | --- | --- | --- | --- | --- |
| Successful inhibition – go | | | | | |
| Negative | -34, -10, -2 | 4.7 | 855 | 6.25x10^-4^ | Left insular and central opercular cortices, putamen, anterior superior temporal gyrus, precentral gyrus, inferior frontal gyrus pars opercularis |
| Failed inhibition – go | | | | | |
| Positive | -36, 16, -28 | 4.28 | 438 | 2.68x10^-2^ | Left temporal pole, anterior parahippocampal gyrus |
| Positive | 22, 16, 2 | 4.38 | 428 | 3.04x10^-2^ | Right putamen |
| Failed – successful inhibition | | | | | |
| Positive | -58, 8, 12 | 4.15 | 2033 | 1.79x10^-7^ | Left precentral gyrus, inferior frontal gyrus pars opercularis, insular and central opercular cortices, putamen, anterior superior temporal gyrus, temporal pole, anterior parahippocampal gyrus |
| Positive | 8, 2, 6 | 3.81 | 609 | 1.01x10^-2^ | Right caudate, thalamus, putamen, pallidum |

Supplementary Table 4 The associations between behavioral correlates of response inhibition and ADHD symptoms, controlling for PRS-ADHD.

|  | Regressing dependent variable on independent variable and mediator | | | | | | | |
| --- | --- | --- | --- | --- | --- | --- | --- | --- |
|  | Independent variable (IV): PRS PT1 | | | | Mediator (M): MRT | | | |
| Dependent variable | β (SE) | p-uncor | p-FDR | R^2^-IV | β (SE) | p-uncor | p-FDR | R^2^-M |
| Total symptoms | 0.21 (0.047) | 1.35x10^-5^ | 4.05x10^-5^ | 0.042 | 0.146 (0.048) | 0.002 | 0.002 | 0.019 |
| IA symptoms | 0.189 (0.047) | 6.83x10^-5^ | 1.02x10^-4^ | 0.034 | 0.121 (0.047) | 0.01 | 0.01 | 0.013 |
| HI symptoms | 0.207 (0.047) | 1.67x10^-5^ | 4.01x10^-5^ | 0.041 | 0.141 (0.048) | 0.003 | 0.003 | 0.016 |
|  | Independent variable (IV): PRS PT1 | | | | Mediator (M): IRT | | | |
| Dependent variable | β (SE) | p-uncor | p-FDR | R^2^-IV | β (SE) | p-uncor | p-FDR | R^2^-M |
| Total symptoms | 0.194 (0.046) | 3.13x10^-5^ | 6.26x10^-5^ | 0.038 | 0.277 (0.048) | 1.87x10^-8^ | 1.12x10^-7^ | 0.063 |
| IA symptoms | 0.178 (0.046) | 1.2x10^-4^ | 1.6x10^-4^ | 0.032 | 0.231 (0.048) | 2.02x10^-6^ | 8.08x10^-6^ | 0.043 |
| HI symptoms | 0.188 (0.046) | 5.2x10^-5^ | 8.91x10^-5^ | 0.035 | 0.278 (0.048) | 1.71x10^-8^ | 2.05x10^-7^ | 0.059 |

Supplementary Table 5 The associations between neural correlates of response inhibition and ADHD symptoms, controlling for PRS-ADHD.

|  | Regressing dependent variable on independent variable and mediator | | | | | | | |
| --- | --- | --- | --- | --- | --- | --- | --- | --- |
|  | Independent variable (IV): PRS PT1 | | | | Mediator (M): Left fronto-insular regions and putamen (Successful inhibition – go) | | | |
| Dependent variable | β (SE) | p-uncor | p-FDR | R^2^-IV | β (SE) | p-uncor | p-FDR | R^2^-M |
| Total symptoms | 0.238 (0.048) | 1.12x10^-6^ | 1.12x10^-5^ | 0.05 | 0.072 (0.045) | 0.116 | 0.183 | 0.005 |
| IA symptoms | 0.213 (0.047) | 8.88x10^-6^ | 2.96x10^-5^ | 0.041 | 0.062 (0.044) | 0.163 | 0.233 | 0.004 |
| HI symptoms | 0.233 (0.048) | 2.19x10^-6^ | 1.64x10^-5^ | 0.048 | 0.049 (0.046) | 0.286 | 0.343 | 0.003 |
|  | Independent variable (IV): PRS PT1 | | | | Mediator (M): Left temporal pole and anterior PHG (Failed inhibition – go) | | | |
| Dependent variable | β (SE) | p-uncor | p-FDR | R^2^-IV | β (SE) | p-uncor | p-FDR | R^2^-M |
| Total symptoms | 0.239 (0.048) | 9.01x10^-7^ | 1.12x10^-5^ | 0.051 | -0.081 (0.046) | 0.082 | 0.145 | 0.007 |
| IA symptoms | 0.213 (0.047) | 7.74x10^-6^ | 2.9x10^-5^ | 0.041 | -0.07 (0.045) | 0.124 | 0.189 | 0.005 |
| HI symptoms | 0.242 (0.048) | 7.43x10^-7^ | 1.12x10^-5^ | 0.052 | -0.096 (0.046) | 0.04 | 0.075 | 0.01 |
|  | Independent variable (IV): PRS PT1 | | | | Mediator (M): Right putamen (Failed inhibition – go) | | | |
| Total symptoms | 0.212 (0.048) | 1.07x10^-5^ | 3.21x10^-5^ | 0.042 | 0.05 (0.046) | 0.28 | 0.343 | 0.002 |
| IA symptoms | 0.185 (0.047) | 9.31x10^-5^ | 2x10^-4^ | 0.032 | 0.073 (0.045) | 0.107 | 0.178 | 0.005 |
| HI symptoms | 0.22 (0.048) | 6.47x10^-6^ | 2.77x10^-5^ | 0.044 | 0.007 (0.046) | 0.875 | 0.883 | 1x10^-4^ |
|  | Independent variable (IV): PRS PT1 | | | | Mediator (M): Left fronto-insular, putamen, anterior temporal regions, and PHG (Failed – successful inhibition) | | | |
| Total symptoms | 0.229 (0.047) | 3.83x10^-6^ | 2.3x10^-5^ | 0.046 | -0.026 (0.047) | 0.577 | 0.641 | 0.001 |
| IA symptoms | 0.201 (0.048) | 3.44x10^-5^ | 7.94x10^-5^ | 0.036 | -0.007 (0.046) | 0.883 | 0.883 | 1x10^-4^ |
| HI symptoms | 0.228 (0.049) | 4.93x10^-6^ | 2.47x10^-5^ | 0.045 | -0.022 (0.047) | 0.636 | 0.681 | 7x10^-4^ |
|  | Independent variable (IV): PRS PT1 | | | | Mediator (M): Right basal ganglia and thalamus (Failed – successful inhibition) | | | |
| Total symptoms | 0.209 (0.048) | 1.69x10^-5^ | 4.22x10^-5^ | 0.04 | 0.053 (0.046) | 0.257 | 0.335 | 0.002 |
| IA symptoms | 0.185 (0.047) | 1.06x10^-4^ | 2.12x10^-4^ | 0.032 | 0.061 (0.046) | 0.182 | 0.248 | 0.003 |
| HI symptoms | 0.215 (0.049) | 1.27x10^-5^ | 3.46x10^-5^ | 0.041 | 0.028 (0.047) | 0.555 | 0.64 | 4x10^-4^ |

Supplementary Table 6 The associations between behavioral correlates of response inhibition and ADHD symptoms.

|  | Regressing dependent variable on mediator | | | | | | | | | | | |
| --- | --- | --- | --- | --- | --- | --- | --- | --- | --- | --- | --- | --- |
|  | Dependent variable | | | | | | | | | | | |
|  | Total symptom score (N=423) | | | | Inattention symptoms (N=438) | | | | Hyperactivity-impulsivity symptoms (N=431) | | | |
| Independent variable | β (SE) | p-uncor | p-FDR | R^2^-IV | β (SE) | p-uncor | p-FDR | R^2^-IV | β (SE) | p-uncor | p-FDR | R^2^-IV |
| MRT | 0.182 (0.048) | 1.84x10^-4^ | 4.14x10^-4^ | 0.029 | 0.154 (0.047) | 0.001 | 1.5x10^-3^ | 0.021 | 0.179 (0.048) | 2.62x10^-4^ | 4.72x10^-4^ | 0.026 |
| IRT | 0.307 (0.049) | 1.02x10^-9^ | 4.59x10^-4^ | 0.078 | 0.257 (0.048) | 1.92x10^-7^ | 5.76x10^-7^ | 0.053 | 0.311 (0.049) | 7.04x10^-10^ | 4.59x10^-9^ | 0.079 |
| SSRT | 0.091 (0.048) | 0.056 | 6.3x10^-2^ | 0.006 | 0.118 (0.046) | 0.011 | 0.014 | 0.011 | 0.055 (0.048) | 0.25 | 0.25 | -0.001 |

Supplementary Table 7 The associations between neural correlates of response inhibition and ADHD symptoms.

|  | Regressing dependent variable on mediator | | | | | | | | | | | |
| --- | --- | --- | --- | --- | --- | --- | --- | --- | --- | --- | --- | --- |
|  | Dependent variable | | | | | | | | | | | |
|  | Total symptom score (N=423) | | | | Inattention symptoms (N=438) | | | | Hyperactivity-impulsivity symptoms (N=431) | | | |
| Independent variable | β (SE) | p-uncor | p-FDR | R^2^-IV | β (SE) | p-uncor | p-FDR | R^2^-IV | β (SE) | p-uncor | p-FDR | R^2^-IV |
| Left fronto-insular regions and putamen (Successful inhibition – go) | 0.025 (0.045) | 0.589 | 0.648 | 7x10^-4^ | 0.023 (0.044) | 0.605 | 0.648 | 6x10^-4^ | 0.001 (0.046) | 0.983 | 0.983 | -0.001 |
| Left temporal pole and anterior PHG (Failed inhibition – go) | -0.03 (0.047) | 0.518 | 0.648 | 0.001 | -0.03 (0.045) | 0.508 | 0.648 | 0.001 | -0.043 (0.047) | 0.357 | 0.578 | 0.002 |
| Right putamen (Failed inhibition – go) | 0.085 (0.046) | 0.067 | 0.251 | 0.006 | 0.105 (0.045) | 0.021 | 0.205 | 0.01 | 0.048 (0.047) | 0.303 | 0.578 | 0.002 |
| Left fronto-insular, putamen, anterior temporal regions, and PHG (Failed – successful inhibition) | 0.04 (0.046) | 0.385 | 0.578 | 0.001 | 0.046 (0.045) | 0.314 | 0.578 | 0.002 | 0.048 (0.047) | 0.304 | 0.578 | 0.002 |
| Right basal ganglia and thalamus (Failed – successful inhibition) | 0.095 (0.046) | 0.041 | 0.205 | 0.008 | 0.096 (0.045) | 0.034 | 0.205 | 0.009 | 0.073 (0.047) | 0.12 | 0.36 | 0.005 |
